# Measurements of daily energy intake and total energy expenditure in people with dementia in care homes: The use of wearable technology

**DOI:** 10.1007/s12603-017-0870-y

**Published:** 2017-01-17

**Authors:** Jane Murphy, J. Holmes, C. Brooks

**Affiliations:** Bournemouth University, Faculty of Health & Social Sciences, Christchurch Road, Bournemouth, BH1 3LT UK

**Keywords:** Dementia, energy expenditure, energy intake, technology, diet, care home, activity

## Abstract

**Objective:**

To estimate daily total energy expenditure (TEE) using a physical activity monitor, combined with dietary assessment of energy intake to assess the relationship between daily energy expenditure and patterns of activity with energy intake in people with dementia living in care homes.

**Design and setting:**

A cross-sectional study in care homes in the UK.

**Participants:**

Twenty residents with confirmed dementia diagnosis were recruited from two care homes that specialised in dementia care.

**Measurements:**

A physical activity monitor (Sensewear™ Armband, Body Media, Pittsburgh, PA) was employed to objectively determine total energy expenditure, sleep duration and physical activity. The armband was placed around the left upper triceps for up to 7 days. Energy intake was determined by weighing all food and drink items over 4 days (3 weekdays and 1 weekend day) including measurements of food wastage.

**Results:**

The mean age was 78.7 (SD ± 11.8) years, Body Mass Index (BMI) 23.0 (SD ± 4.2) kg/m^2^; 50% were women. Energy intake (mean 7.4; SD ± 2.6) MJ/d) was correlated with TEE (mean 7.6; SD ± 1.8 MJ/d; r=0.49, p<0.05). Duration of sleeping ranged from 0.4-12.5 (mean 6.1) hrs/d and time spent lying down was 1.3-16.0 (8.3) hrs/d. On average residents spent 17.9 (6.3-23.4) hrs/d undertaking sedentary activity. TEE was correlated with BMI (r=0.52, p<0.05) and body weight (r=0.81, p<0.001) but inversely related to sleep duration (r=-0.59, p<0.01) and time lying down (r=-0.62, p<0.01). Multiple linear regression analysis revealed that after taking BMI, sleep duration and time spent lying down into account, TEE was no longer correlated with energy intake.

**Conclusions:**

The results show the extent to which body mass, variable activity and sleep patterns may be contributing to TEE and together with reduced energy intake, energy requirements were not satisfied. Thus wearable technology has the potential to offer realtime monitoring to provide appropriate nutrition management that is more person-centred to prevent weight loss in dementia.

## Introduction

The numbers and proportion of older adults living with dementia are increasing with an aging population worldwide. The World Health Organisation reports that 7.7 million new cases are identified each year with 65.7 million people diagnosed by 2030, that represents a near doubling since 2010 (1). In the United Kingdom, there are about 820,000 people with dementia, that is costing the economy more than £23 billion per year (2, 3) with 80% of people living in care homes having some form of dementia or cognitive decline.

Nutritional problems, especially unexplained weight loss is often observed in dementia which leads to loss of muscle mass and strength, increased risk of falls, functional dependence and reduced quality of life (4-7). The mechanisms inducing weight loss include inadequate energy intake to meet requirements, higher resting energy expenditure, raised physical activity or a combination of these factors (7-8). There have been limited studies using doubly labelled water and indirect calorimetry to understand the aetiology of wasting but no compelling evidence to support the notion that a hypermetabolic state contributes to weight loss (7). It is also likely that agitation, perambulation and immobility in individuals with dementia can lead to weight loss and waste energy while trying to perform daily tasks combined with difficulty in eating and ingesting less food (9).

As dementia progresses and even from early stages, a reduction in dietary intake has been shown in people with dementia living in nursing homes (10-11). Consequently accelerated weight loss often occurs due to dietary problems that operate against a background of associated behavioural disorders. These include food aversions, impaired appetite, agitation, exaggerated activity and wandering behaviour as well as the physical difficulties to take food to the mouth, chewing and swallowing (12). The reason for a lowered food intake are not understood but changes in the brain regions involved in hunger control, a declining sense of smell and taste (13) and an earlier satiety due to a greater sensitivity to cholecystokinin are all potential contributors (7). Taken together, these findings indicate that the relationship between weight loss, malnutrition and cognitive function is complex and thus poor nutritional status is likely to be both a cause and consequence of dementia.

Establishing individual daily energy needs in people with dementia can be problematic. If based solely on measures of intake alone, there are potential inaccuracies associated with calculating energy intakes from recorded diaries or interviews and recalls and the gross assumption that the individual is in energy balance. Moreover current regression equations to predict energy needs for older people fail to account for the diversity of disease state, body composition and activity (14).

Thus, there is a need to identify new strategies and approaches to monitor and better manage nutritional status for those living with dementia and prevent the consequences of weight loss and cognitive decline. The application of wearable technology offers new possibilities for the nutritional management of dementia. The feasibility of using accelerometer monitoring devices to measure physical activity has been shown in community-living older people with cognitive impairment (15). The light weight physical activity monitor (Sensewear™ Armband, Body Media, Pittsburgh, PA) can be used to objectively determine measures related to physical activity and sleep but also total energy expenditure (TEE). The device measures tri-axial acceleration, skin temperature, galvanic skin response and heat flux. It has been shown to be valid in resting, exercise and free-living conditions and its application for self-monitoring with real-time feedback has been reported (16-20). The accuracy of TEE and activity energy expenditure using this activity monitor against criterion methods has been demonstrated (21).

The aim of the present study was to use accelerometry combined with dietary assessment of energy intake to assess the relationship between daily energy expenditure and patterns of activity with energy intake in a group of people with dementia living in care homes.

## Methods

### Design

This cross-sectional study was performed between July 2014 and October 2014. Care home residents were recruited from two care homes in South West England that specialised in dementia care with confirmed clinical diagnosis of dementia (assessed by Mini Mental State Examination with scores less than 23; 22). Recruitment was restricted to two care homes owing to the intensive nature of the study that has taken a whole systems approach that involves everyone who has responsibility for delivering food and nutrition including managers, health care professionals, care and catering staff as well as relatives and family members. Residents with dementia were selected and recruited under the informed guidance of the care home managers. Exclusion criteria were residents with advanced dementia (advanced deterioration of language and cognition), those receiving palliative care treatment or artificial enteral or parenteral nutrition, dysphagia leading to aspiration, mood disorders and aggressive and volatile behaviour which would make it difficult to perform the measurements.

### Ethics

Ethical approval for the study was provided through Bournemouth University Research Ethics Committee. Written consent was provided by the care home manager for access to the care home population. Residents provided informed verbal and/or written consent for their involvement in the study as well as a close family member and the care managers of both care homes. No identifiable information was collected from any of the residents. Confidentiality and anonymity was ensured by the use of numbers to code and represent data and all data was securely stored in locked environments.

### Data collection and procedures

All care home staff who were responsible for the delivery of food and drink (caregiver, chef, kitchen assistant) were trained by a researcher (Registered Nutritionist JH) on how to record all food and drink intake. Total dietary intake from all food and drink consumed was collected over 5 days by care home staff using a weighed plate-wastage method to improve the accuracy of the intake, using electronic weighing scales (Salter, Tonbridge). Each food item was weighed prior to serving (to the nearest 1.0g). Weighing each item of food as it was served on the plate ensured the appearance of the meal remained and therefore would not interfere with residents’ enjoyment of consumption. In both nursing homes, food was prepared in the care home kitchen and meals were weighed out by the kitchen staff and then delivered by heated food trolley or directly, depending on proximity to the kitchen to the eating area at tables for usually 4 people. Some residents preferred to eat alone in their rooms. The care home staff served the meals to the residents and helped then with feeding as required. Following consumption, any leftover food items were weighed and recorded individually. Food and drink consumed was calculated from the amount consumed minus wastage. In order to obtain the most accurate results when calculating weight of food consumed, the weight of non-energy yielding drinks e.g. tea and coffee were excluded but the amount and type of milk used, sugar in drinks was included. Some brand food item names and amounts were recorded using household measures such as ‘cup’ and ‘glass’ and standard portion sizes like ‘slice’ and ‘bar’ with the exception of foods and drinks consumed for cooked meals. Recipes and preparation methods were also obtained from the kitchen staff. The mealtimes were typically: breakfast at 9.00 am, lunch at 12.00 pm, supper at 17.00pm. Lunch was the main hot meal of the day. Any additional food, snacks, supplements and drinks were observed and recorded by the care staff including any food or drinks consumed overnight. Residents received their regular meals and no foods or snacks were altered or modified in any way for the purpose of this study.

Information on age, gender, medication use, use of nutritional energy and protein supplements, height and weight was obtained from the care home records. Weight was measured by the same person from the care home on a monthly basis (to the nearest 0.1 kg) with residents wearing normal clothing. For those residents who were unable to stand upright, information on body height was a proxy measure based on length of forearm (23). Body Mass Index (BMI) was calculated as weight in kilograms divided by height in metres squared.

All participants wore the Sensewear™ Armband (Bodymedia, Pittsburgh, PA) for up to 7 days, placed halfway between the acromion and olecranon processes on the upper left arm. The device uses four sensors to assess energy expenditure, sleep duration and sedentary physical activity duration as well as step count. The armband includes a tri-axial accelerometer, a thermistor-based skin sensor, a proprietary heat flux sensor and a galvanic skin response sensor. The recording of all food and drink consumed and use of accelerometers were checked by researchers on a daily basis who addressed any concerns and questions.

### Nutrient analysis

Nutrient intakes of food, drink and snacks were analysed using dietary analysis software (NetWISP v4.0, Tinuviel Software, Warrington, UK). This software employs the most recently updated McCance and Widdowson nutrient databank (24).

### Statistical analyses

All analyses were carried out using SPSS (Version 23.0, Chicago, IL, USA). Data was checked for normal distribution by Shapiro-Wilk test. Descriptive data was presented as mean ± standard deviation (SD). Energy intakes were analysed and the contribution made by carbohydrate, fat and protein was calculated and presented as % energy. Pearson’s Product Moment correlation coefficient (r) was used to assess associations between TEE and independent variables (age, BMI, body weight, energy intake, sleep duration, time lying down and sedentary activity). Spearman’s rank correlation was applied to assess the association between TEE and number of steps per day. The paired student’s t-test compared the difference between TEE and energy intake. Multiple stepwise linear regression models were created to further evaluate the association between TEE and energy intake adjusting for BMI, sleep duration and time lying down. The results were considered statistically significant at the 0.05 level (2-tailed).

## Results

### General Characteristics

There were 22 residents who were recruited to the study. Of these, two residents did not continue with the study due to illness and refusal by one resident to apply the accelerometer correctly. Therefore data collected from 20 residents from the two care homes were used for the analysis. The mean age was 78.7 ± 11.8 years and 50% of the group were women (Table 1). The residents’ mean weight was 67.0 ± 17.0 kg and mean height 169.5 ± 9.7 cm. Mean BMI was 23.0 ± 4.2 kg/m^2^. Six residents were underweight (< 20 kg/m^2^), eight had a BMI within the normal range (20-25 kg/m^2^), five were classified as overweight (> 25 kg/m2) and one resident was obese (30 kg/m^2^) (23, 25). Eight of the residents were prescribed oral energy and protein supplements based on medium to high risk scores using Malnutrition Universal Screening Tool (23).

**Table 1:**
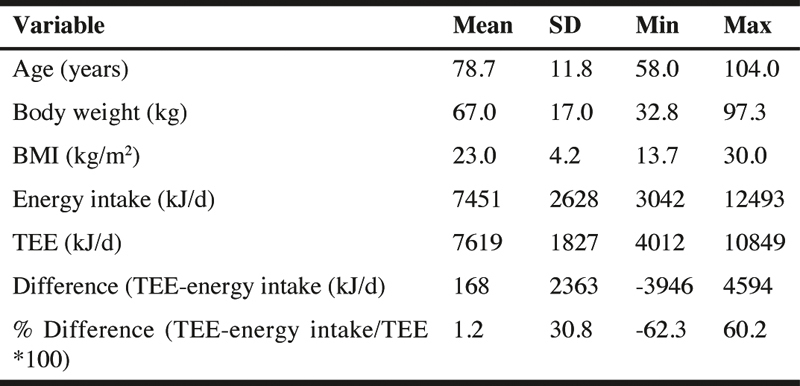
Characteristics, energy intake and total energy expenditure (TEE) of the residents

### Energy and macronutrient intake

The average intake of energy from food, drink and any supplements was 7451 ± 2628 kJ/d with a 4 fold difference observed between residents (3042-12493 kJ/d) (Table 1). The mean intake of total carbohydrate was 205.8 ± 76.9 g/d and accounted for the majority of energy (44 ± 6.7%). For total fat, the mean intake was 87.5 ± 27.9 g/d, providing 40.5 ± 11.9% energy. Whereas mean protein intake was 53.2 ± 19.6 g/d, accounting for 12 ± 2.2 % of energy intake. Energy intake was significantly correlated with body weight (r=0.50, p<0.05).

### Total energy expenditure

TEE was 7619 ± 1827 kJ/d, ranging from 4012-10849 kJ/d. On the assumption that the measurements represent the true habitual level of intake and expenditure, there was no significant difference between the measures of energy intake and TEE (p=0.753) indicating that the group as a whole were essentially in energy balance when studied.

**Figure 1:**
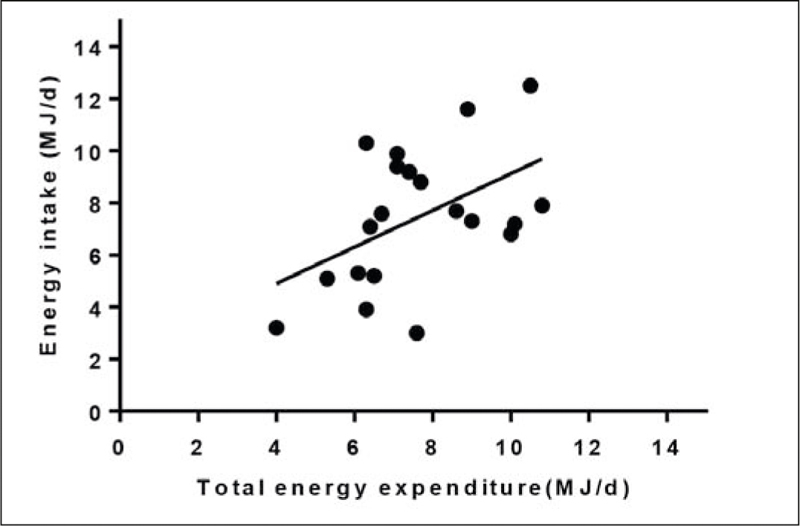
Total energy expenditure and energy intake in care home residents living with dementia

Energy intake was moderately correlated with TEE (r=0.49, p<0.05; Figure 1). The mean difference between TEE and energy intake was 168 ± 2363 kJ/d (p=0.753)and the percentage difference varied from -62.3% to 60.2% (1.2 ± 30.8%; Table 1). There were 11 residents who required between 188 to 4594 kJ/d more energy to meet their requirements (Table 1). Of these, there were four residents who were underweight, two residents in the normal weight range and five residents who were overweight. On the other hand, nine residents were consuming in excess of between 766 to 3946 kJ/d of their measured energy needs. Of these there were two residents who were classified as underweight, five residents who were normal weight, one resident was overweight and one resident who just fell within the obese category.

TEE was positively correlated with BMI (r=0.52, p<0.05) and strongly correlated with body weight (r=0.81, p<0.001). There was no relationship between TEE and age (r=-0.30, p=0.207).

### Sleep and physical activity patterns

Duration of sleeping ranged from 0.4-12.5 (6.1 ± 2.8) hrs/d and time spent lying down was 1.3-16.0 (8.3±3.9) hrs/d. On average residents spent 17.9 ± 4.5 (6.3-23.4) hrs/d undertaking sedentary activity.

**Figure 2:**
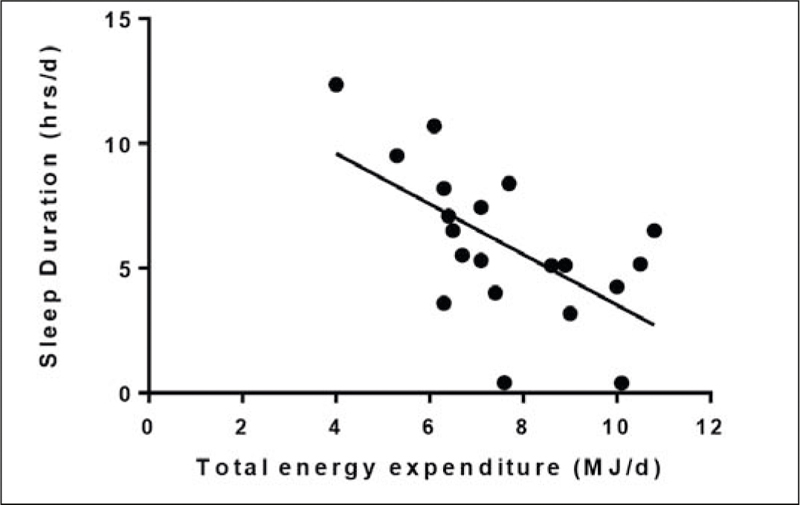
Total energy expenditure and time spent sleeping and in care home residents living with dementia

**Figure 3:**
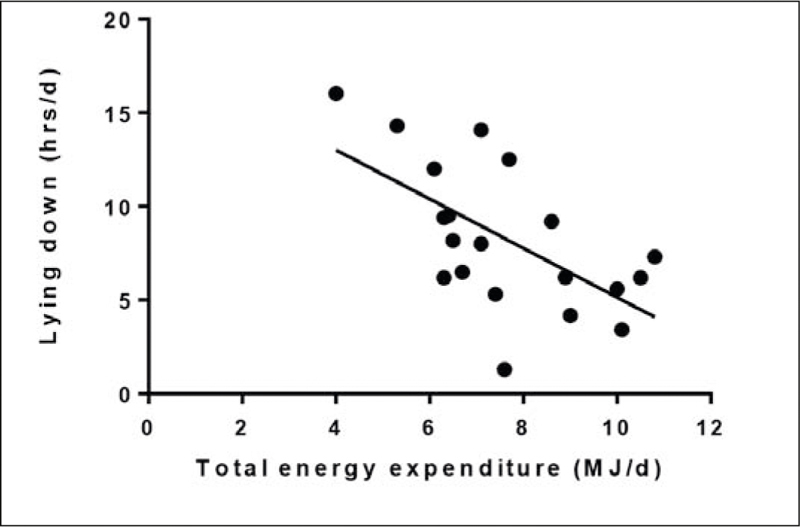
Total energy expenditure and time spent lying down in care home residents living with dementia

Sleep duration was inversely related to TEE (r=-0.59, p<0.01; Figure 2). There was an inverse correlation between time lying down and TEE (r=-0.62, p<0.01; Figure 3). TEE was positively correlated with number of steps per day (r= 0.60, p<0.01). There was no relationship between TEE and sedentary activity (r=0.33, p=0.159) or TEE and age (r=-0.30, p=0.207).

Further analysis using multiple linear regression models showed that TEE was no longer significantly related to energy intake (Table 2) after adjusting for BMI, sleep duration and time lying down and taken together accounted for 57% of the variability in TEE.

## Discussion

This is the first study that has reported objective measurements of TEE using accelerometry combined with measurements of energy intake in people with dementia living in care homes. The results show that the relationship between TEE and energy intake is influenced by body mass, variable activity and sleep patterns that contributed to low energy intakes in some of the residents. Overall the monitoring devices were well received by both the residents and care staff and in all but one resident encountered no problems with their application. Thus the study would support their feasibility and acceptability to use in people with dementia.

As a group, daily energy intakes compared favourably with intakes measured in studies among similarly aged community-dwelling people with dementia (9, 26-27). This observation reflects good practices for delivering food and nutrition including fortification of food, use of supplements, influence of caregiver. As such some residents had intakes that exceeded their requirements yet over half of the cohort were still not meeting their TEE and/or were losing weight due to low intakes. The reasons for this would require closer examination which might be due to altered activity associated with changed behaviours given the observed differences in sleep and sedentary activity patterns. Those residents who had greater TEE were those who required less sleep and/or spent less time lying down. On the other hand, residents were likely to have poor appetites due to reduced activity and as the result of other known behavioural disorders affecting food consumption including food aversions, forgetfulness, sensory changes (13). Another explanation for low energy intake might be attributed to the eating environment and meal experience to promote appropriate nutrition and hydration that has been raised in other studies for people with dementia (28). As such this is an aspect that would require more consideration by the care home staff, to introduce ways to increase food intake. Thus the use of accelerometry has highlighted the need to recognise the importance of good nutrition for people with dementia to prevent weight loss by taking a more person-centred approach.

**Table 2:**
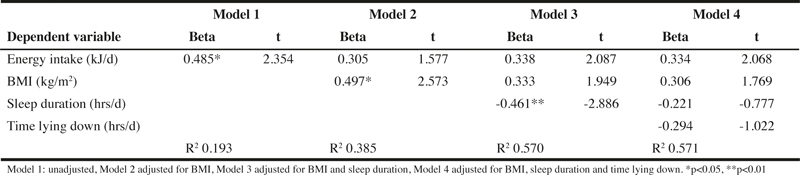
The standardised coefficients of total energy expenditure to energy intake after adjustment for potential confounders using multiple linear regression models

Overall the proportion of energy from fat was greater than current recommendations (no more than 35% of energy from fat; 29) and could account for intakes in excess of TEE in some residents. This was not surprising as the energy density of the food was enhanced through proactive fortification using butter, margarine, whole fat milk in both residential care homes. In spite of fortifying food, there were some residents who were still not receiving adequate energy intake to meet requirements which might be attributed to altered sleeping and activity behaviours associated with advancing dementia.

It is recognised the limitations associated with collecting dietary intake data reliably that can contribute to altered food consumption, incomplete or inaccurate reporting (30). To reduce the influence of underreporting, any inaccuracies and inconsistencies were mitigated by taking a ‘whole systems approach’ that involved focused working with two care homes to ensure that everyone involved in the care of the participants were given thorough training on how to record foods and plate wastage. The collection of dietary intake was closely supervised by the research team during the study period. Twenty residents living with dementia in two care homes were included in the present study and the limitation in sample size is recognised. However given the feasibility in the approaches used, more research is needed using larger sample sizes and with inclusion of some of the observed potential confounding factors as well as longitudinal analysis of other dietary modifiable factors that has potential to influence a decline in cognition (31).

## Conclusion

The current study reports the value of real-time monitoring using wearable technology to measure TEE and the influence of body mass, variable activity and sleep patterns on energy intake in dementia. Thus it demonstrates the importance of assessing TEE in dementia because altered behaviours can influence nutritional status as energy requirements were not satisfied, that could lead to weight loss. Further research needs to be carried out using a large sample size and over the various stages of dementia to understand the complex factors that are contributing to weight loss and cognitive decline. Therefore this information will better inform appropriate nutritional management strategies with more person-centred approaches for people living with dementia in care homes and other care settings.

*Acknowledgements*: The project was funded by the Burdett Trust for Nursing and is gratefully acknowledged. We thank and acknowledge the cooperation of the care homes – in particular the consent from managers and help from the kitchen and front-line care assistants and nursing staff, the residents themselves and their families who gave permission for us to carry out the research without which none of the work would have been possible.

*Conflict of interest*: All the authors declare no conflict of interest.

## References

[1] World Health Organisation. (2012). Dementia: A Public Health Priority. World Health Organisation Geneva, Switzerland.

[2] Alzheimer’s Society Low expectations. (2013). Attitudes on choice, care and community for people with dementia in care homes.

[3] Alzheimer’s Disease International World Alzheimer Report 2013 Journey of Caring. (2014). An Analysis of Long-Term Care for Dementia.

[4] Gillette Guyonnet S, Abellan Van Kan G, Alix E, Andrieu S, Belmin J, Berrut G (2007). IANA (International Academy on Nutrition and Aging) Expert Group: weight loss and Alzheimer’s disease. J Nutr Health Aging..

[5] Gao S, Nguyen JT, Hendri HC, Unverzagt FW, Hake A, Smith-Gamble V (2011). Accelerated weight loss and incident dementia in an elderly African-Ammerican cohort. J Am Geriatr Soc..

[6] Soto ME, Secher M, Gillette-Guyonnet S, Abellan KG, Andrieu S, Nourhashemi F (2012). Weight loss and rapid cognitive decline in community-dwelling patients and Alzheimer’s disease. J Alzheimers Dis..

[7] Sergi G, De Rui M, Coin A, Meral Inelman E, Manzato E (2013). Weight loss and Alzheimer’s disease: temporal and aetiologic connections. Proc Nutr Soc..

[8] Poehlman ET, Dvorak RV. Energy expenditure, energy intake, and weight loss in Alzheimer disease. Am J Clin Nutr. 2000;71:650S-55S. 10.1093/ajcn/71.2.650s 10681274

[9] Galesi LF, Leandro-Merhi VA, de Oliveira MR (2013). Association between indicators of dementia and nutritional status in institutionalised older people. Int J Older People Nurs..

[10] Suominen M, Laine T, Routasalo P, Pitkala KH, Rasanen L (2004). Nutrient content of served food, nutrient intake and nutritional status of residents with dementia in a Finnish nursing home. J Nutr Health Aging..

[11] Nijs KA, de Graaf C, Kok FJ, van Staveren WA (2006). Effect of family style mealtimes on quality of life, physical performance, and body weight of nursing home residents: cluster randomised controlled trial. BMJ..

[12] Alzheimer’s Disease International. (2014). Nutrition and dementia. A review of available research..

[13] Morley JE (2001). Decreased food intake with aging. J Gerontol A Biol Sci Med..

[14] Starling RD, Poehlman ET (2000). Assessment of energy requirements in elderly population. Eur J Clin Nutr.

[15] Erickson KI, Barr LL, Weinstein AM, Banducci AM, Akl SL, Santo NM (2013). Measuring Physical Activity Using Accelerometry in a Community Sample With Dementia. J Am Geriatr Soc..

[16] Shuger SL, Barry VW, Sui X, McClain A, Hand GA, Wilcox S, Meriwether RA, Hardin JW, Blair SN (2011). Electronic feedback in a diet-and physical activity-based lifestyle intervention for weight loss: a randomized controlled trial. Int J Behav Nutr Phys Act..

[17] Anderson AS, Craigie AM, Caswell S, Treweek S, Stead M, Macleod M (2014). The impact of a bodyweight and physical activity intervention (BeWEL) initiated through a national colorectal cancer screening programme: randomised controlled trial. BMJ..

[18] Fruin ML, Rankin JW (2004). Validity of a multi-sensor armband in estimating rest and exercise energy expenditure. Med Sci Sports Exerc..

[19] Welk G M, Clain JJ, Eisenmann JC, Wickel EE (2007). Field validation of the MTI Actigraph and BodyMedia armband monitor using the IDEEA monitor. Obesity (Silver Spring)..

[20] St-Onge M, Mignault D, Allison DB, Rabasa-Lhoret R (2007). Evaluation of a portable device to measure daily energy expenditure in free-living adults. Am J Clin Nutr..

[21] Mackey DC, Manini TM, Schoeller DA, Koster A, Glynn NW, Goodpaster BH (2011). Validation of an armband to measure daily energy expenditure in older adults. J Gerontol A Biol Sci Med Sci.

[22] Folstein MF, Folstein SE, McHugh PR (1975). Mini-mental state, a practical method for grading the cognitive state of patients for the clinician. J Psychiatr Res.

[23] Elia M (2003). Screening for Malnutrition: A Multidisciplinary Responsibility. Development and Use of the Malnutrition Universal Screening Tool (‘MUST’) for Adults.

[24] Food Standards AgencyPublic Health England (2014). McCance and Widdowson’s The Composition of Foods.

[25] World Health Organization. (2000). Obesity: preventing and managing the global epidemic. WHO Technical Report Series, No. 894 World Health Organization Geneva, Switzerland.

[26] De Bruin SR, Oosting SJ, Tobi H, Blauw YH, Schols J D, Groot CP (2010). Day care at green care farms: a novel way to stimulate dietary intake of community-dwelling older people with dementia. J Nutr Health Aging..

[27] Engelheart S, Akner G (2015). Dietary intake of energy, nutrients and water in elderly people living at home or in nursing home. J Nutr Health Aging..

[28] Keller HH, Martin LS, Dupuis S, Reimer H, Genoe R (2015). Strategies to Support Engagement and Continuity of Activity During Mealtimes for Families Living with Dementia; a Qualitative Study. BMC Geriatr..

[29] Department of Health. (2002). Care Homes for Older People National Minimum Standards.

[30] Prentice RL, Mossavar-Rahmani Y, Huang Y, Van Horn L, Beresford SA (2011). Evaluation and comparison of food records, recalls, and frequencies for energy and protein assessment by using recovery biomarkers. Am J Epidemiol..

[31] Handing EP, Small BJ, Reynolds SL, Kumar NB (2015). Impact of dietary factors and inflammation on cognition among older adults. J Prev Alz Dis.

